# Acquisition of Multiple Prior Distributions in Tactile Temporal Order Judgment

**DOI:** 10.3389/fpsyg.2012.00276

**Published:** 2012-08-14

**Authors:** Yasuhito Nagai, Mayu Suzuki, Makoto Miyazaki, Shigeru Kitazawa

**Affiliations:** ^1^Department of Neurophysiology, Graduate School of Medicine, Juntendo UniversityBunkyo-ku, Tokyo, Japan; ^2^Research Institute for Time Studies, Yamaguchi UniversityYamaguchi, Japan; ^3^Dynamic Brain Network Group, Graduate School of Frontier Biosciences, Osaka UniversitySuita, Osaka, Japan; ^4^Department of Brain Physiology, Graduate School of Medicine, Osaka UniversitySuita, Osaka, Japan

**Keywords:** Bayesian estimation, eye movement, prior distribution, tactile, temporal order judgment, visual cue

## Abstract

The Bayesian estimation theory proposes that the brain acquires the prior distribution of a task and integrates it with sensory signals to minimize the effect of sensory noise. Psychophysical studies have demonstrated that our brain actually implements Bayesian estimation in a variety of sensory-motor tasks. However, these studies only imposed one prior distribution on participants within a task period. In this study, we investigated the conditions that enable the acquisition of multiple prior distributions in temporal order judgment of two tactile stimuli across the hands. In Experiment 1, stimulation intervals were randomly selected from one of two prior distributions (biased to right hand earlier and biased to left hand earlier) in association with color cues (green and red, respectively). Although the acquisition of the two priors was not enabled by the color cues alone, it was significant when participants shifted their gaze (above or below) in response to the color cues. However, the acquisition of multiple priors was not significant when participants moved their mouths (opened or closed). In Experiment 2, the spatial cues (above and below) were used to identify which eye position or retinal cue position was crucial for the eye-movement-dependent acquisition of multiple priors in Experiment 1. The acquisition of the two priors was significant when participants moved their gaze to the cues (i.e., the cue positions on the retina were constant across the priors), as well as when participants did not shift their gazes (i.e., the cue positions on the retina changed according to the priors). Thus, both eye and retinal cue positions were effective in acquiring multiple priors. Based on previous neurophysiological reports, we discuss possible neural correlates that contribute to the acquisition of multiple priors.

## Introduction

Signals in our nervous system are noisy (van Beers et al., 2002). To achieve precise perception and behavior based on noisy signals, our brain has to overcome sensory signal variability. Recent studies have shown that our brain makes the most of our prior knowledge or experience in estimating a parameter of concern in a manner comparable to Bayesian estimation, which theoretically minimizes expected errors in estimation (Kersten et al., 2004; Knill and Pouget, 2004; Kording and Wolpert, 2006; Wolpert, 2007). Bayesian estimation has been shown to occur during error estimation in reaching (Kording and Wolpert, 2004; Tassinari et al., 2006), force reproduction (Kording et al., 2004), timing estimation (Miyazaki et al., 2005; Jazayeri and Shadlen, 2010), and temporal order judgment (TOJ) of two tactile stimuli, where one is delivered to each hand (Miyazaki et al., 2006).

**Figure 1:**
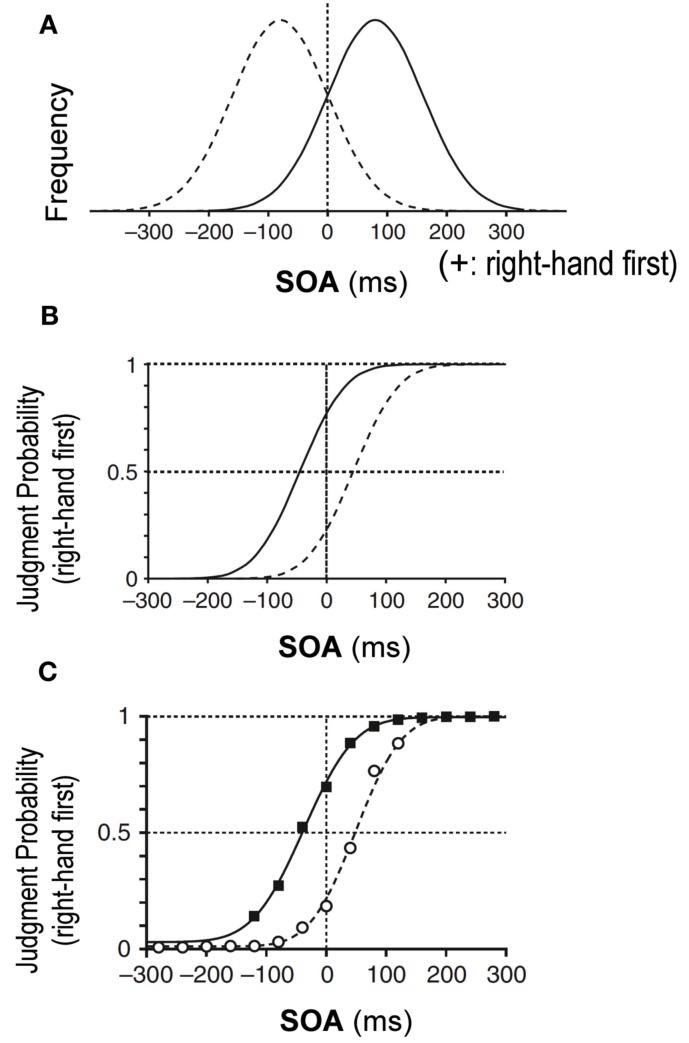
**Bayesian estimation in temporal order judgment (TOJ) of two tactile stimuli, one delivered to each hand (Miyazaki et al., 2006)**. **(A)** Prior distributions of stimulus-onset asynchrony (SOA) of the two stimuli. On the *x*-axis, positive SOA values signify that the right hand was stimulated before the left, and negative values signify that the left hand was stimulated first. The *y*-axis is the SOA frequency. The solid Gaussian has a mean (μ_prior_) of +80 ms and a SD (σ_prior_) of 80 ms. Given this prior, subjects were exposed to stimulus pairs with the right hand stimulated first in ∼84% of trials. Conversely, the dashed Gaussian (μ_prior_ = −80 ms, σ_prior_ = 80 ms) shows that subjects were exposed to stimulus pairs with the left hand earlier in ∼84% of trials. **(B)** Predictions of the TOJ responses by the Bayesian estimation model. The psychometric functions shift away from peaks of the prior distributions. **(C)** Experimental observation for the tactile TOJ. The psychometric functions of the subjects’ responses were in accordance with the Bayesian estimation model.

According to the Bayesian estimation theory, an estimated parameter is shifted toward the most frequent value or to the peak of the prior distribution that has been learned through repeated past experiences. Let us assume, for example, that the stimulation interval of two tactile stimuli, one delivered to each hand, was sampled from a Gaussian distribution biased toward “right-hand first” stimuli (Figure 1A, solid Gaussian). Then, according to the Bayesian estimation theory, simultaneous delivery of two stimuli would lead to an estimated interval shifted toward the positive value, or a “right-hand first” interval, which would result in a larger probability of “right-hand first” judgment. Owing to the shift toward the “right-hand first” judgment, the overall psychometric function would shift to the left, away from the peak of the prior distribution (Figure 1B, solid curve). Indeed, we previously observed that a psychometric function shifted away from the peak of the prior distribution as predicted from the Bayesian estimation theory (Figure 1C) (Miyazaki et al., 2006).

In earlier studies that reported Bayesian estimation, parameters to be estimated were sampled from a single prior distribution within a task period. After a certain period of exposure to the prior distribution, participants were tested on whether their estimation shifted, as predicted from the Bayesian estimation theory. Kording and Wolpert (2004) showed that subjects can adapt their estimate to a distribution with two peaks, but still the prior distribution was single. However, in our daily life, it is not reasonable to assume that we always encounter a single prior distribution. Rather, the distribution of a parameter may differ in a context-dependent manner. Therefore, we asked whether we are able to simultaneously acquire two prior distributions and examined what types of signals may serve as cues that enable us to discriminate one distribution from another.

In Experiment 1, we used color cues (condition 1). We chose color cues because human and monkey subjects have been shown to adapt to two opposing force fields in a reaching task when a different color is presented for each (Wada et al., 2003; Osu et al., 2004; Yamamoto et al., 2007). In two other conditions, we used body posture cues in addition to color cues because different body postures served as effective cues to acquire two movement skills under different force fields (Gandolfo et al., 1996). In condition 2, we used eye movements, and participants looked at the top or bottom target according to the color cues. In condition 3, participants opened or closed their mouths according to the color cues. As a result, we found that participants were able to acquire two prior distributions when eye movement cues were used (condition 2). In Experiment 2, we further tested which of two parameters that change with eye movements, eye position, or cue position on the retina was critically important for acquiring the two distributions.

## Materials and Methods

### Participants

Fifty-one healthy volunteers (29 men and 22 women; age, 18–25 years; 50 right-handed and 1 left-handed) participated in the study. Thirty participants were randomly allocated to Experiment 1 (10 for each of the three conditions), and 20 to Experiment 2 (10 for each of the two conditions). One participant was additionally assigned to Experiment 2 (condition 2) owing to an outlier in the data. All participants were naïve to the purpose of the experiments. Approval of the study was granted by the ethics committee of Juntendo university, and all participants gave written informed consent in accordance with institutional guidelines.

### Apparatus and general task procedures

**Figure 2:**
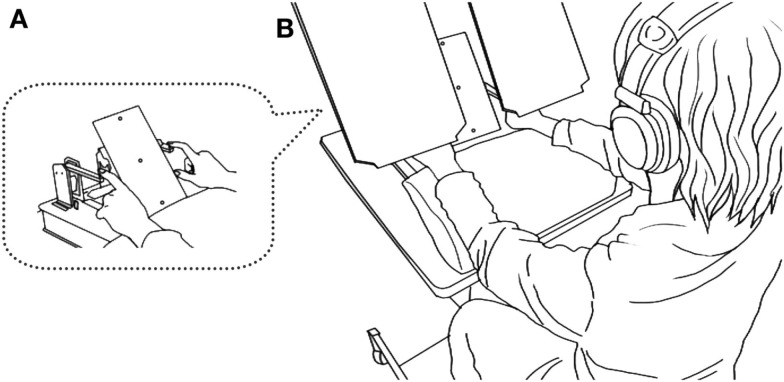
**Overview of the experimental setup**. A seated participant placed the pads of both index fingers on a pair of piezoelectric skin contacts **(A)**. The contacts delivered brief mechanical pulses, one to each hand. White noise was played through headphones placed over the participants’ plugged ears to shut out sounds and ensure only tactile sensations from the contacts. Participants reported the side of the first stimulus by pressing one of two foot pedals corresponding to the stimulated side. A two-color LED (central LED) placed in front of the participants’ eyes (∼40 cm away) was illuminated (red or green) 1 s before the tactile stimuli in each trial. Participants opened their eyes and viewed the LED, but boards placed between their eyes and hands prevented them from viewing their hands **(B)**.

Participants sat in a chair, rested their arms comfortably on a urethane foam pad, and touched the skin contacts with their index fingers (Figure 2A). They judged the order of successive tactile stimuli delivered through the contactors, one to each hand, and reported the side of the first stimulus by pressing one of two foot pedals in a forced choice manner.

The skin contacts were placed 20 cm apart, one 10 cm to the right and the other 10 cm to the left of the midline of the body. Each contact was driven by a multilayer piezoelectric actuator (T.I.K., Japan), to which a rectangular voltage pulse (64 V, 2 ms) was applied to produce a small and short-lasting movement of the contact in the direction perpendicular to the skin surface. The stimulus was at least 10 times as large as the threshold in terms of the applied voltage. To mask the sound from the piezoelectric devices, white noise (∼90 dB SPL) was played through headphones placed over the participants’ ears, and the participants also wore earplugs. The participants kept their eyes open throughout the experiments, but a direct view of their hands and the contacts was blocked by placing a pair of boards over each hand (Figure 2B).

At the beginning of each trial, each participant was asked to fixate his or her eyes on a two-color light emitting diode (central LED) that was placed 40 cm away from the participants. Two additional red LEDs were placed above and below the central LED at a distance of 10 cm (∼14° in the visual angle). Then, the central LED was illuminated in red or green in Experiment 1, or either the top or bottom LED was illuminated in Experiment 2 for 3 s. The illumination started 1 s prior to the onset of tactile stimuli, and the five groups of participants followed group-specific instructions according to the color cues (Groups 1–3) or spatial cues (Groups 4 and 5). They continued to fixate on the central LED (Groups 1 and 5), made a saccade to the top or bottom target (Groups 2 and 4), or opened or shut their mouth (Group 3). Each instructed movement was completed before the tactile stimulus delivery, and the participant was required to maintain their eye or mouth position during the delivery and until they had executed a response on the appropriate foot pedal. After each response, the participants resumed the initial position. Each experiment consisted of 800 trials. To maintain a high level of alertness, the subjects were given a short rest every 80 trials.

Experimenters monitored the face and eye/mouth movements of each participant through a video camera on a 14″TV monitor during the experiments, to confirm that each participant was faithfully following our instruction. The monitored images were recorded in video tapes. Some parts of the records were lost in accident; however, the remaining records of 7836 trials over 12 participants were examined by three recruited raters (3402 trials over 6 participant for condition 2 of Experiment 1, 686 trials over 1 participant for condition 3 of Experiment 1, and 3748 trials over 5 participants for condition 2 of Experiment 5). For condition 2 of Experiment 1 (with eye movements according to the color cues), participants failed to make correct eye movements in 57 of 3402 trials. The mean error rate was 1.7% (95% confidence interval, CI: 1.2–2.1%). For condition 3 of Experiment 1 (with mouth movements according to the color cues), the error rate was 0.7% (5/686, 95% CI: 0.1–1.4%). For condition 2 of Experiment 2 (spatial cues without eye movements), participants erroneously made eye movements from the center LED in 10 of 3748 trials (the mean error rate: 0.3%, 95%CI: 0.1–0.4%). Though we were only able to examine a part of the data, the error rate was negligibly small in that exclusion of 2.1% of data at random (the maximum of the 95% CI) little affected the main results. In addition, we confirmed in the video that participants did not make any apparent head movements, while they attended to the visual cues, though some showed a gradual drift of a few centimeters over each block of 80 trials.

### Association between the cues and the prior distributions

In half of the trials with one of the two-color cues (Experiment 1) or one of the two spatial cues (Experiment 2), the stimulus onset asynchronies (SOAs) were sampled from a Gaussian distribution that was biased toward a right-hand-first asynchrony by +80 ms, with a standard deviation (SD) of 80 ms (Figure 1A, solid Gaussian). The means and the SD were identical to those used in our previous study (Miyazaki et al., 2006). Here, positive intervals indicated that the right hand was stimulated first. More precisely, SOAs were randomly assigned from 11 intervals (−120, −80, −40, 0, +40, +80, +120, +160, +200, +240, and +280 ms) with different frequencies (5, 10, 25, 50, 70, 80, 70, 50, 25, 10, and 5 trials) in the 400 trials with the cue in each experiment. In these 400 trials, the right hand was stimulated earlier than the left hand in 78% (310/400) of the trials, and simultaneously in 13% (50/400) of the trials. We regard the Gaussian distribution as the right-hand-first prior distribution. In the other half of the trials with the other cue, SOAs were sampled from another Gaussian distribution that was centered on −80 ms so that the left hand was stimulated earlier in most trials (Figure 1A, dashed Gaussian, left-hand-first prior distribution). The association between the cues (red/green or top/bottom) and the prior distribution was counterbalanced across participants. One of the two cues was presented for each trial in random order. It is worth noting that the participants were not informed of the correlation between the cue and the prior distribution.

#### Experiment 1 (color cues with or without movements)

The purpose of Experiment 1 was to test whether participants were able to acquire the two different priors according to the color cues (red/green) without any additional movements (condition 1), with eye movements (condition 2), and with mouth movements (condition 3). Thirty participants were randomly allocated to one of the three conditions (10 participants for each).

#### Experiment 2 (spatial cues with or without eye movements)

Based on positive results in condition 2 (color cues with eye movements) of Experiment 1, in which not only the eye position but also the cue position on the retina changed, we further tested which of the two, the eye position or the cue position on the retina, was responsible for acquiring the two priors. To achieve this, we provided participants with spatial cues, and participants made a saccade to the illuminated LED (condition 1) or kept fixating on the central LED (condition 2). In condition 1, eye position changed with the cue staying in the center of the retina. In condition 2, the cue position changed, but the eye position did not. Twenty participants were randomly allocated to one of the two conditions (10 participants for each). One participant was additionally assigned to condition 2 because an outlier was detected after data analysis.

### Analysis

For each condition in each experiment, the response data were sorted according to the prior distribution, then according to the SOA in order to calculate order judgment probabilities that the right hand was stimulated earlier than the left for each prior distribution. We then fitted the order judgment probabilities by a cumulative density function of a Gaussian distribution as a function of the SOA:

(1) $$p\left(t\right)=\overset{t}{\underset{-\infty }{\int }}G\left(\tau ;d,\sigma \right)d\tau ,$$

where

(2) $$G\left(\tau ;d,\sigma \right)=\frac{1}{\sqrt{2\pi \;\sigma }}\exp\left(\frac{-{\left(\tau -d\right)}^{2}}{2\sigma^{2}}\right).$$

In these equations, *t*, τ, *d*, and σ denote the SOA, a parameter representing time, size of the horizontal transition (i.e., the point of subjective simultaneity, PSS), and the temporal resolution, respectively. MATLAB (optimization tool box) was used for fitting by adjusting *d* and σ to maximize the log-likelihood. This analysis was applied to the data from individual subjects as well as the data pooled over 10 subjects for each condition.

The key parameter was the PSS (*d*). As explained in the Section “Introduction,” the PSS with the overall psychometric function shifts away from the peak of each prior distribution: to the left for the right-hand-first prior and to the right for the left-hand-first prior (Figure 1B). Therefore, assuming Bayesian estimation, the PSS for the left-hand-first prior should be larger than the PSS for the right-hand-first prior. Thus, we subtracted the PSS for the left-hand-first prior from that for the right-hand-first prior (PSS difference). We tested the prediction from the Bayesian model that the PSS difference is larger than zero by applying the Wilcoxon signed-rank test to the data from 10 participants for each condition. One outlier (>3 SDs) was excluded from the statistical test in Experiment 2 (condition 2). Multiple comparisons were corrected for each experiment using a Bonferroni correction.

## Results

### Experiment 1 (color cues with or without eye/mouth movements)

**Figure 3:**
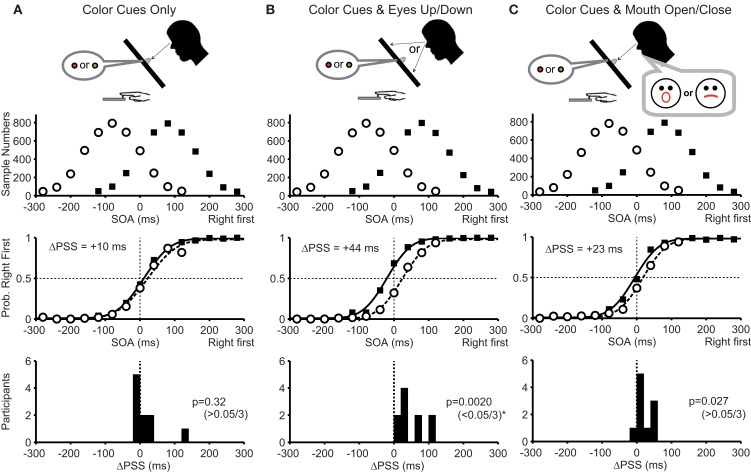
**Experimental conditions and results of Experiment 1**. **(A)** With color cues without any movements (condition 1). **(B)** With color cues and eye movements (condition 2). **(C)** With color cues and mouth movements (condition 3). The top panels show prior distributions biased to the right-hand first (filled squares) and the left-hand first (open circles). The ordinate represents the sample number for each stimulus onset asynchrony (SOA) summed across all participants. The middle panels show the mean probability that the participants judged their right hand as being stimulated first as a function of the SOAs. Note the shifts of two psychometric functions in **(B)** away from the peaks of two prior distributions as predicted by the Bayesian estimation theory. Separation between two psychometric functions, or the difference between the two points of subjective simultaneity (ΔPSS), is shown in each panel. The bottom panels show the number of participants as a function of the PSS difference calculated for each participant. The Bayesian estimation theory predicts positive PSS differences. The *P*-value of the Wilcoxon signed-rank test, under the null hypothesis that the median was zero, is shown in each panel. The level of significance was set to 0.05/3 (Bonferroni correction).

In condition 1, participants fixated on the central color cue position throughout the task. The PSS difference for the pooled data was +10 ms and was slightly in the direction predicted by the Bayesian estimation theory (Figure 3A, middle panel). However, the PSS difference from 10 participants did not reach statistical significance (Figure 3A bottom, median = +2.5 ms; *P* = 0.32). Thus, the color cues alone were not sufficient for the participants to acquire two prior distributions.

In condition 2, participants moved their eyes to the top or bottom target according to the color cue. The PSS difference for the pooled data was +44 ms and was clearly in the direction expected from the Bayesian estimation theory (Figure 3B, middle panel). Accordingly, the PSS difference from 10 participants was significantly larger than zero (Figure 3B bottom, median = +34 ms; *P* = 0.0020 < 0.05/3). Thus, the eye position changes according to the color cues were sufficient for acquiring two prior distributions.

In condition 3, participants opened or closed their mouths according to the color cues. In this condition, we used mouth movements to test whether any movements other than the eye movements enables participants to acquire two prior distributions. The PSS difference for the pooled data was +23 ms and was in the direction expected from the Bayesian estimation theory (Figure 3C, middle panel), but the PSS difference from 10 participants did not reach statistical significance after Bonferroni correction (Figure 3C bottom, median = +12 ms; *P* = 0.027 > 0.05/3).

The results in Experiment 1 demonstrate that the color cues presented in the center of the retina were not sufficient. But, by imposing upward and downward eye movements according to the color cues, the PSS clearly shifted away from the peak of each prior distribution as predicted by the Bayesian estimation theory. It may be argued that saliency to the subject was the key difference. However, the addition of mouth movements, which were as demanding as the eye movement, did not significantly enhance the acquisition of the two prior distributions. This excludes the saliency hypothesis and highlights the importance of eye movements in acquiring the two cue-contingent priors. However, it may still be argued that it was not the eye movement *per se*, but the change in the position of the illuminated LED on the retina that was responsible for the separate acquisition of the two prior distributions. To distinguish between the two possibilities, we introduced spatial cues with and without eye movements in Experiment 2.

### Experiment 2 (spatial cues with or without eye movements)

**Figure 4:**
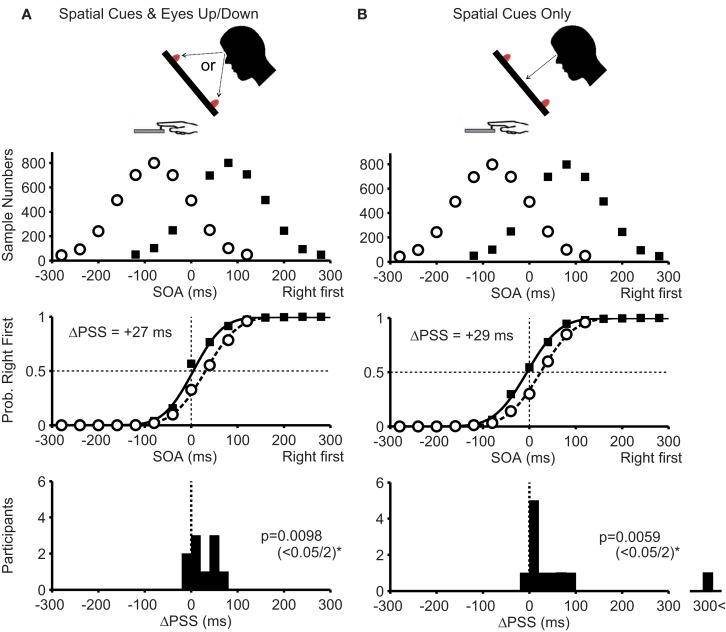
**Experimental conditions and results of Experiment 2**. **(A)**: With spatial cues and eye movements (condition 1). **(B)**: With spatial cues without eye movements (condition 2). In condition 1, the participants’ eye positions changed, but the cue positions on their retinas were constant. Conversely, in condition 2, the participants’ eye positions were constant, but the cue positions on their retinas were changed. In the bottom panels, the level of significance was set to 0.05/2 (Bonferroni correction). Note that an outlier (ΔPSS = 370 ms), shown in the bottom panel in **(B)**, was excluded from calculating the mean response curve and calculating the *P*-value. Other conventions are as in Figure 3.

In condition 1, the participants looked at the illuminated LED at the top or bottom (Figure 4A, top). Therefore, eye position changed according to the spatial cues, but the position of the illuminated target stayed in the center of the retina. The PSS difference for the pooled data was +27 ms in the direction predicted by the Bayesian estimation theory (Figure 4A, middle panel). The PSS difference from 10 participants was significantly larger than zero (Figure 4A bottom, median = +21 ms; *P* = 0.0098 < 0.05/2).

In condition 2, the participants kept fixating on the central LED when a spatial cue was presented at the top or bottom (Figure 4B, top). Therefore, the eye position was unchanged, but the position of the illuminated LED changed on the retina. In this condition, the PSS difference for the pooled data was +29 ms in the direction expected from the Bayesian estimation theory (Figure 4B, middle panel). The PSS difference from 10 participants was significantly larger than zero (Figure 4B, bottom, median = +20 ms; *P* = 0.0059 < 0.05/2).

The results in Experiment 2 show that both eye position and cue position on the retina served as effective cues to separately acquire two prior distributions.

## Discussion

In this study, we investigated whether subjects could simultaneously acquire two prior distributions in tactile TOJ and examined what types of signals may serve as cues that enable the discrimination of one prior distribution from another. In Experiment 1, different colored LEDs (red and green) were associated with two prior distributions of the SOA: one biased toward the right-hand-first and the other toward the left-hand-first SOAs. The results from condition 1 demonstrated that color cue alone did not yield different TOJ responses. In condition 2, when participants shifted their gazes to the top or bottom target in response to the color cues, they generated two different TOJ responses according to the two prior distributions, as predicted by the Bayesian estimation theory. In condition 3, when participants opened or closed their mouths according to the color cues, they did not distinguish between the two prior distributions. The results show that body postures do not necessarily serve as effective cues. The lack of significant discrimination in condition 3 further shows that a color cue is ineffective, even if it is attended by the participants. Taken together, human participants were able to acquire two prior distributions in parallel when the two distributions were associated with different eye positions.

However, at this stage, we cannot fully conclude that the change in the eye position was responsible for the acquisition of the two prior distributions because the cue position on the retina also changed in conjunction with eye movement. The possibility remained that the acquisition of the two prior distributions was independent of the eye position but dependent on the cue position on the retina. Therefore, Experiment 2 was designed to examine this possibility. In this experiment, each prior distribution was associated with one of the two LED illuminations above or below the fixation point. In the first condition, participants shifted their gaze to the illuminated LED so that the eye position changed, but the cue remained in the center of their retinas. In the second condition, participants continued to gaze at the fixation point so that the cue positions on the retina changed without changing the eye position. We observed that participants were able to respond differently as predicted by the Bayesian estimation theory in both conditions. These results show that the human brain can acquire two prior distributions depending on eye position and the spatial cue position on the retina.

In our study, color cues were not effective for acquiring two prior distributions. The results may seem to contradict with previous findings that participants adapted to two opposing force fields in reaching tasks with color cues (Wada et al., 2003; Osu et al., 2004; Yamamoto et al., 2007). The discrepancy may be explained by the difference in the tasks: a TOJ task that did not involve motor control in ours, and reaching tasks that required control of arm movements in the others. However, Gandolfo et al. (1996) reported that color cues were ineffective for acquiring arm movement skills under two different force fields. Thus the involvement of motor control cannot be a critical factor. Another possibility may be the salience of color cues. This does not seem to apply, either, because a flood of colored light, a very salient color cue, was not effective in Gandolfo et al. (1996). The third possibility may be the difference in the ways of presenting two conditions. Osu et al. (2004) reported that the color cue was not effective when two cues alternated, but was effective when either cue was presented at random. This was consistent with the finding that the color cue was effective when the cue was presented at random (Wada et al., 2003), and another finding that the color cue was ineffective when the cues alternated in blocks (Gandolfo et al., 1996). However, the color cue was effective, even when two cues alternated in blocks (Yamamoto et al., 2007). In addition, the color cue was not effective in our case, even though they were chosen at random. Thus the way of presenting two cues, at random or in an alternate order, does not explain the discrepancies, either. We cannot provide a definite suggestion at this stage, as to why the effect of color cues varies across different studies.

Where in the brain are the prior distributions stored and retrieved depending on the eye and cue positions? With regard to the storage site of stimulation intervals, responsible areas should receive signals from the bilateral hand stimuli. Previous studies with monkeys determined that the convergence of bilateral hand signals occurs in the upper bank of the intraparietal sulcus (Iwamura et al., 1994), and many neurons in the superior parietal cortex are driven by the stimulation of either hand (Duffy and Burchfiel, 1971; Sakata et al., 1973). As for eye position and cue-position signals, retinotopic receptive fields of many neurons in the inferior parietal lobule, specifically the lateral intraparietal area and area 7a, change systematically with gaze angle (Andersen et al., 1985, 1990; Read and Siegel, 1997). These monkey studies demonstrate that necessary information including signals from bilateral hands, eye position signals, and retinotopic receptive fields converge in the parietal lobule. In addition, the inferior parietal lobule has been implicated in TOJs of tactile signals (Takahashi et al., 2012). Thus, it is likely that the parietal association cortex plays an essential role in the acquisition and retrieval of prior distributions for tactile TOJ depending on the eye- and cue-position signals.

However, it is worth noting that many areas other than the parietal lobule have been implicated in tactile TOJ, such as the bilateral posterior part of the middle temporal gyri, the bilateral premotor cortices, and the bilateral inferior frontal gyri (Takahashi et al., 2012). Thus, it is also likely that the storage and the retrieval of prior distributions are achieved through dynamic interactions between these distant cortical areas. Further studies are required to examine these possibilities.

To conclude, we have clearly shown that participants were able to acquire two prior distributions in tactile TOJs, when appropriate cues were provided. We have also shown that either eye position or cue position on the retina served as an effective cue. The novel findings would certainly provide clues for investigating neural basis of Bayesian estimation.

## Conflict of Interest Statement

The authors declare that the research was conducted in the absence of any commercial or financial relationships that could be construed as a potential conflict of interest.
